# Genome-wide identification of the WRKY gene family in *Codonopsis pilosula* and functional analysis of *CpWRKY8* under drought stress

**DOI:** 10.3389/fpls.2026.1961042

**Published:** 2026-09-30

**Authors:** Shiyi Song, Meiqi Liu, Meiqi Zhang, Shan Jiang, Shuangshuang Shan, Weichao Ren, Weili Liu, Huiyan Cao, Xiubo Liu, Wei Ma

**Affiliations:** 1 College of Pharmacy, Heilongjiang University of Chinese Medicine, Harbin, Heilongjiang, China; 2 College of Life Science, Northeast Forestry University, Harbin, Heilongjiang, China; 3 Heilongjiang Vocational Institute of Ecological Engineering, Harbin, Heilongjiang, China

**Keywords:** *Codonopsis pilosula*, *CpWRKY8*, drought stress, transcriptional regulation, WRKY TF

## Abstract

*Codonopsis pilosula* (Franch.) Nannf., a perennial herbaceous species in the family Campanulaceae, is widely used in traditional medicine for its dried roots. Drought stress severely constrains both the yield and the accumulation of bioactive compounds in this species. In this study, we performed a genome-wide identification and phylogenetic analysis of the WRKY TF (TF) family in *C. pilosula*, identifying 49 members that were classified into seven subfamilies. Among them, *CpWRKY8*, a member of the Group IIa subfamily, localized to the nucleus and showed transcriptional activation activity in yeast. Under drought stress conditions, *Nicotiana tabacum* plants overexpressing (OE) *CpWRKY8* exhibited greater tolerance to drought stress than wild-type (WT) *N. tabacum* plants, as evidenced by higher survival rates, lower malondialdehyde (MDA) levels, and higher superoxide dismutase (SOD), peroxidase (POD), and catalase (CAT) activities; furthermore, these physiological advantages were largely maintained following rehydration. Furthermore, yeast one-hybrid (Y1H) and dual-luciferase reporter (DLR) assays demonstrated that the abscisic acid (ABA) receptor CpPYR/PYL activated the *CpWRKY8* promoter in heterologous systems. In summary, we propose a working model in which CpPYR/PYL indirectly upregulates *CpWRKY8*; this module enhances drought tolerance by increasing antioxidant capacity, thereby providing a theoretical basis for the molecular breeding of drought-tolerant medicinal plants.

## 1 Introduction

*C. pilosula* is a perennial herb belonging to the genus *Codonopsis* in the family Campanulaceae. Its dried roots are rich in active constituents such as polysaccharides, saponins and alkaloids, and it is one of the most widely used medicinal herbs in clinical practice (Xie et al., 2020; Chu et al., 2024; Guo et al., 2024; Kaixian et al., 2025). The traditional production areas for this species are predominantly in the arid and semi-arid regions of Shanxi and Gansu provinces (Xiao-jun et al., 2017; Ge, 2022; Liu, 2023; Bowen et al., 2024). Studies have shown that moderate drought can significantly promote the accumulation of active compounds in the roots, suggesting that drought may positively activate its secondary metabolic pathways (Liang et al., 2021; Wang et al., 2024, Wang et al., 2025, Wang et al., 2026b). Although WRKY transcription factors (TFs) have been implicated in secondary metabolism in several medicinal plants, their roles in *C. pilosula* remain largely unknown, and the present study focuses on the drought-responsive function of *CpWRKY8*. Understanding the molecular mechanisms underlying drought responses in *C. pilosula* may provide insights into the potential relationship between stress adaptation and medicinal quality formation.

TFs function as central switches and regulators within the complex signaling networks that govern plant responses to drought stress (Hu et al., 2022; Khoso et al., 2022; Wei et al., 2024). WRKY TFs constitute one of the largest TF families in plants and are characterized by a highly conserved WRKYGQK domain and a typical zinc-finger motif. They are extensively involved in regulating plant growth, development, and signal transduction in response to various environmental stresses (Eulgem and Somssich, 2007; Javed and Gao, 2023; Angidi and Razzaq, 2026). Extensive studies have demonstrated the crucial roles of WRKY TFs in drought stress responses. For instance, *AdWRKY40* from *Arachis duranensis* enhances drought tolerance by regulating reactive oxygen species (ROS) pathways and temperature-dependent signaling (Li et al., 2026a). Silencing *TaWRKY24* in *Triticum aestivum* significantly reduces the drought tolerance of tobacco and wheat seedlings (Yu et al., 2023). *AtWRKY40* in *Arabidopsis thaliana* mitigates drought-induced damage by modulating antioxidant capacity and osmotic balance (Che et al., 2018). Furthermore, *GhWRKY17* in *Gossypium hirsutum* mediates drought and salt stress responses through ABA signaling and ROS homeostasis (Yan et al., 2014). Despite these advances in model and crop species, a comprehensive genome-wide characterization and functional analysis of the WRKY family in *C. pilosula* has been lacking, significantly hindering our understanding of its stress response mechanisms.

Furthermore, the perception and transduction of drought signals often depend on signaling cascades mediated by the core phytohormone ABA (Aslam et al., 2022, Aslam et al., 2025). In the canonical ABA signaling pathway, ABA is perceived by the PYR/PYL/RCAR receptors (Park et al., 2009; Lu et al., 2025), which inhibit type 2C protein phosphatases (PP2Cs), leading to the activation of sucrose non-fermenting 1-related protein kinase 2 (SnRK2) kinases. Subsequently, activated SnRK2s phosphorylate downstream TFs such as AREB/ABF, which in turn initiate the expression of stress-responsive genes (Grill and Himmelbach, 1998; Ma et al., 2009; Park et al., 2009). Recent studies have revealed intricate crosstalk between the ABA signaling network and WRKY-mediated transcriptional regulation. For instance, *AtWRKY18* and *AtWRKY40* are rapidly induced by ABA (Chen et al., 2010). The chloroplast ABA receptor ChlH/ABAR physically interacts with AtWRKY18/40/60 to co-regulate ABA-responsive genes (Shang et al., 2010). Additionally, *TaWRKY2* and *TaWRKY19*, as well as *OsWRKY71* in *Oryza sativa*, have been shown to participate in ABA-mediated drought stress responses (Niu et al., 2012; Bataller et al., 2024). However, in *C. pilosula*, the question of how upstream ABA receptors regulate downstream WRKY TFs through signaling cascades to mediate drought responses and secondary metabolite accumulation remains unanswered.

In this context, we used whole-genome data of *C. pilosula* to systematically identify members of the WRKY gene family and characterize their roles under drought stress. Building on this, we focused on the TF *CpWRKY8* and, through subcellular localization, analysis of transcriptional activation activity and heterologous overexpression experiments in *N. tabacum*, investigated three specific questions: (1) genome-wide identification and phylogenetic analysis of the *CpWRKY* gene family; (2) the function of *CpWRKY8* in *N. tabacum* under drought stress; (3) the upstream regulatory mechanisms governing *CpWRKY8* transcription. Our findings further suggest a potential regulatory connection between CpPYR/PYL-associated ABA signaling and *CpWRKY8* transcription. In summary, this study proposes a working model of “ABA-CpPYR/PYL signaling cascade-*CpWRKY8* transcriptional response”. This discovery not only provides key genetic resources and a theoretical foundation for an in-depth analysis of the drought resistance mechanisms and quality formation in *C. pilosula*, but also offers new strategies for molecular-assisted breeding of authentic medicinal herbs in arid and semi-arid regions.

## 2 Materials and methods

### 2.1 Plant materials and growth conditions

*C. pilosula* seeds were purchased from Dingxi City, Gansu Province (35°36′29″N 104°35′31″E) and sown at the Medicinal Plant Garden of Heilongjiang University of Traditional Chinese Medicine. Two-month-old *C. pilosula* seedlings were selected for subsequent experiments. *Nicotiana benthamiana* grown in a 1:1 (v/v) mixture of soil and vermiculite and *N. tabacum* grown on Murashige and Skoog (MS) solid medium (HB8469-7, Haibo Biotechnology, China) were cultured under constant conditions (25°C, photoperiod of 16 hours light/8 hours dark). Five-to seven-week-old *N. benthamiana* and one-month-old sterile *N. tabacum* seedlings were used for further assays.

### 2.2 Identification, chromosomal localization, and physicochemical characterization of CpWRKY family members

The genomic data for *C. pilosula* (PRJNA1068481) were downloaded from the NCBI database (https://www.ncbi.nlm.nih.gov/Structure/bwrpsb/bwrpsb.cgi), while the *A. thaliana* WRKY protein sequences were obtained from the TAIR database (https://www.arabidopsis.org/). Using the AtWRKY protein sequences as references, a sequence similarity search against the *C. pilosula* proteome was performed using TBtools (Chen et al., 2023), with an E-value threshold of 1e-5. Candidate sequences were further verified for the presence of a complete WRKY domain using PlantTFDB (https://planttfdb.gao-lab.org/), the Pfam online tool (http://pfam.xfam.org/search#tabview=tab0), and NCBI-CDD (https://www.ncbi.nlm.nih.gov/Structure/bwrpsb/bwrpsb.cgi). The chromosomal localization of WRKY family genes in *C. pilosula* was determined using the NCBI online database; a chromosomal localization map of *CpWRKY* genes was constructed using TBtools; genes were renamed based on their chromosomal positions, and a chromosomal distribution map was plotted. Using AtWRKY proteins as references, multiple sequence alignment was performed with TBtools. Physicochemical properties, including molecular weight, isoelectric point, instability coefficient, and hydrophilicity, were predicted using the ExPASy online tool; subcellular localization was predicted using the WoLFPSORT website (https://wolfpsort.hgc.jp/).

### 2.3 Phylogenetic, conserved motif, and gene structure analyses of CpWRKY members

The identified members were classified into seven subfamilies according to the classification criteria established for *A. thaliana* (Wang et al., 2011). The WRKY protein sequences of *C. pilosula* and *A. thaliana* were aligned using MUSCLE, and poorly aligned regions were trimmed with trimAl. A maximum likelihood (ML) phylogenetic tree was constructed using IQ-TREE v2.2.2.6 implemented in TBtools (Chen et al., 2023). The best-fit amino acid substitution model (JTT+I+G4) was automatically determined by ModelFinder (Kalyaanamoorthy et al., 2017) based on the Bayesian Information Criterion (BIC). Branch support was assessed with 5 000 ultrafast bootstrap replicates (Hoang et al., 2018). The resulting tree was visualized and annotated using the Interactive Tree of Life (iTOL) online tool (https://itol.embl.de/) (Letunic and Bork, 2021). Conserved domains were identified using the NCBI CD-Search tool. Conserved motifs were analyzed using the MEME suite (version 5.0.5, http://meme-suite.org/tools/meme) with the number of motifs set to 10 and an E-value threshold of 1e-10. The phylogenetic tree, motif distribution, and conserved domains were integrated and visualized using TBtools.

### 2.4 Promoter cis-acting element and synteny analyses

The 2 000 bp upstream promoter sequences of the *CpWRKY* genes were retrieved, and cis-regulatory elements (CREs) were predicted using the PlantCARE database (http://bioinformatics.psb.ugent.be/webtools/plantcare/html/). The distribution of elements was visualized using TBtools. Genome data for *A. thaliana* (GCA_000005425.2), *O. sativa* (GCA_001433935.1), and *Solanum lycopersicum* (GCF_000188115.4) were downloaded from the NCBI database. The genome data for *Platycodon grandiflorus* were obtained from the Figshare database (https://info.figshare.com/). Synteny analysis within the *C. pilosula* genome and between species (with *A. thaliana*, *O. sativa*, *S. lycopersicum*, and *P. grandiflorus*) was performed using the MCScanX tool implemented in TBtools, with the BLASTP E-value threshold set to 1e-10 for homologous gene pair identification.

### 2.5 Bioinformatic analysis of *CpWRKY8*

The physicochemical properties, secondary structure (SOPMA), tertiary structure (SWISS-MODEL), transmembrane domains (TMHMM Server v. 2.0), signal peptides (SignalP 6.0), phosphorylation sites (NetPhos 3.1a), and N-glycosylation sites (NetNGlyc 1.0) of CpWRKY8 were predicted using the corresponding online servers. The amino acid composition was analyzed using DNAMAN. A maximum likelihood (ML) phylogenetic tree of CpWRKY8 and WRKY40 orthologs was constructed using TBtools.

### 2.6 Subcellular localization assay

The CDS sequence of *CpWRKY8* was cloned from the cDNA of *C. pilosula*, and the coding sequence was fused into the pCAMBIA1300-35S-EGFP vector. The recombinant plasmid 35S::CpWRKY8-EGFP and the empty vector control (35S::EGFP) were co-transfected into *N. benthamiana* leaves via *Agrobacterium tumefaciens* (GV3101 (pSoup-p19))-mediated transformation. Following infiltration, plants were incubated at 25°C in darkness for 24 h and then transferred to a 12 h light period before fluorescence observation. Fluorescence signals were observed using a confocal laser scanning microscope (Zeiss LSM 800). DAPI was used as a nuclear localization signal (Chi et al., 2026). The primer sequences are listed in **Supplementary Table S1**.

### 2.7 Transcriptional activation activity assay

The CDS sequence of *CpWRKY8* was cloned into the pGBKT7 vector to generate the recombinant plasmid pGBKT7-CpWRKY8, with the empty pGBKT7 vector used as a negative control. The recombinant plasmid was transformed into Y2HGold chemically competent cells. The transformed yeast strains were cultured on SD/-Trp medium at 30°C for 3 days and then plated on SD/-Trp and SD/-Trp/-His/-Ade/X-α-gal media for selection and observation (Zhou et al., 2022). The primer sequences are listed in **Supplementary Table S1**.

### 2.8 Generation of *CpWRKY8*-overexpressing *N. tabacum* and drought tolerance analysis

The 35S::CpWRKY8-EGFP recombinant plasmid was introduced into *N. tabacum* via *A. tumefaciens* (GV3101 (pSoup-p19))-mediated leaf disc transformation (Yang et al., 2017). Hygromycin-resistant transgenic lines were identified by PCR. Quantitative real-time PCR (qRT-PCR) was performed using Vazyme qPCR enzyme (Q711-02, Vazyme, China) with *NtActin* as the internal reference gene. The relative expression level of *CpWRKY8* was calculated using the 2^−ΔΔCT^ method (Livak and Schmittgen, 2001). The thermal cycling program was as follows: 95°C for 3 min; 40 cycles of 95°C for 10 s and 60°C for 30 s; and a final step of 95°C for 30 s, 60°C for 1 min, and 95°C for 30 s. The three transgenic lines with the highest expression levels were selected for further analysis. For the seed germination assay, seeds from WT and the three transgenic lines were sown on 1/2 MS medium, 1/2 MS medium supplemented with 25 mg/L hygromycin (Hyg), and 1/2 MS medium containing 200 mM D-mannitol. Each plate was divided into four sectors, with 25 seeds per line, and germination was recorded on day 7. For the seedling drought tolerance assay, WT and transgenic line seeds were germinated on 1/2 MS medium for 10 days and then transplanted into pots containing a 1:1 (v/v) mixture of soil and vermiculite. After normal irrigation until the stage with 4 to 6 leaves, water was withheld for natural drought treatment (Li et al., 2023b), followed by rewatering for 7 days. Plant phenotypes were observed, and samples were collected at appropriate times for physiological and biochemical analyses. For the post-germination growth assay, WT and OE seeds were germinated on 1/2 MS medium with or without 25 mg/L Hyg for 3 days (radicle emergence). They were then transferred to 1/2 MS medium supplemented with 200 mM D-mannitol and cultured vertically for 10 days to observe growth performance (Li, 2025). Samples collected from the seedling drought tolerance assay were used to measure SOD, POD, and CAT activities, as well as MDA content, following the manufacturer’s protocols (Suzhou Grace Biotechnology Co., Ltd.). Three biological replicates were performed for each sample, and data are presented as mean ± standard deviation (SD). The primer sequences are listed in **Supplementary Table S1**.

### 2.9 Yeast one-hybrid (Y1H) assay

The CDS sequence of *CpPYR/PYL* genes (designated *CpPYR/PYL*1 through *CpPYR/PYL*4) were cloned into the pGADT7-Rec2 vector to generate the recombinant plasmids pGADT7-Rec2-CpPYR/PYL1–4. The *CpWRKY8* promoter sequence (defined as the ~2 kb genomic region immediately upstream of the ATG start codon) was cloned into the pHIS2 vector to generate the recombinant plasmid pHIS2-*CpWRKY8.* The pHIS2-*CpWRKY8* and pGADT7-Rec2-CpPYR/PYL1–4 recombinant constructs were transformed into Y187 competent yeast cells. The transformed yeast strains were cultured on SD/-Trp-Leu medium at 30°C for 3 days and then transferred to SD/-Trp-Leu and SD/-Trp-Leu-His medium supplemented with 115 mM 3-amino-1,2,4-triazole (3-AT) (CA1311, Coolaber, China) for selection and observation (Chi et al., 2026). The primer sequences are listed in **Supplementary Table S1**.

### 2.10 Dual-luciferase reporter (DLR) assay

The CDS sequence of *CpPYR/PYL* (designated *CpPYR/PYL*1 through *CpPYR/PYL*4) were cloned into the pGreenII 62-SK vector to generate the effector constructs pGreenII 62-SK-*CpPYR/PYL*1–4. The *CpWRKY8* promoter sequence (defined as the ~2 kb genomic region immediately upstream of the ATG start codon) was cloned into the pGreenII 0800-LUC vector to generate the reporter construct pGreenII 0800-LUC-CpWRKY8. The effector and reporter constructs were co-transformed into *N. benthamiana* leaves via *A. tumefaciens* (GV3101 (pSoup-p19)) mediated infiltration. After infiltration, plants were incubated at 25°C in the dark for 24 h, followed by 12 h of light. Leaves were then sprayed with D-luciferin potassium salt (CL6930, Coolaber, China) and incubated at 37°C for 5 min. Luminescence signals were captured using an *in vivo* plant imaging system (GS-5 000P, Jingyi Technology, China) (Yang et al., 2025). The primer sequences are listed in **Supplementary Table S1**.

## 3 Results

### 3.1 Chromosomal localization and physicochemical properties of CpWRKY family members

Based on the whole-genome data of *C. pilosula*, a total of 49 WRKY gene family members were identified. Chromosomal localization analysis revealed that these genes were unevenly distributed across eight chromosomes and one unanchored contig (Contig260) (**Figure 1**). According to their chromosomal positions, the 49 genes were renamed *CpWRKY1* through *CpWRKY49*, and their basic information and physicochemical properties are summarized in **Table 1**. Sequence analysis showed that the CpWRKY proteins ranged from 103 to 986 amino acids in length, with molecular weights ranging from 12.4 to 107.9 kDa and pI values from 4.87 to 9.99. With the exception of CpWRKY13, CpWRKY42 and CpWRKY49, which are predicted to be stable proteins, the remaining members are all unstable proteins. All members exhibited negative GRAVY values, indicating their hydrophilic nature. Subcellular localization prediction indicated that most CpWRKY proteins were targeted to the nucleus, with the exception of CpWRKY1 (chloroplast), CpWRKY15 (peroxisome), and CpWRKY42 (cytoplasm).

**Figure 1:**
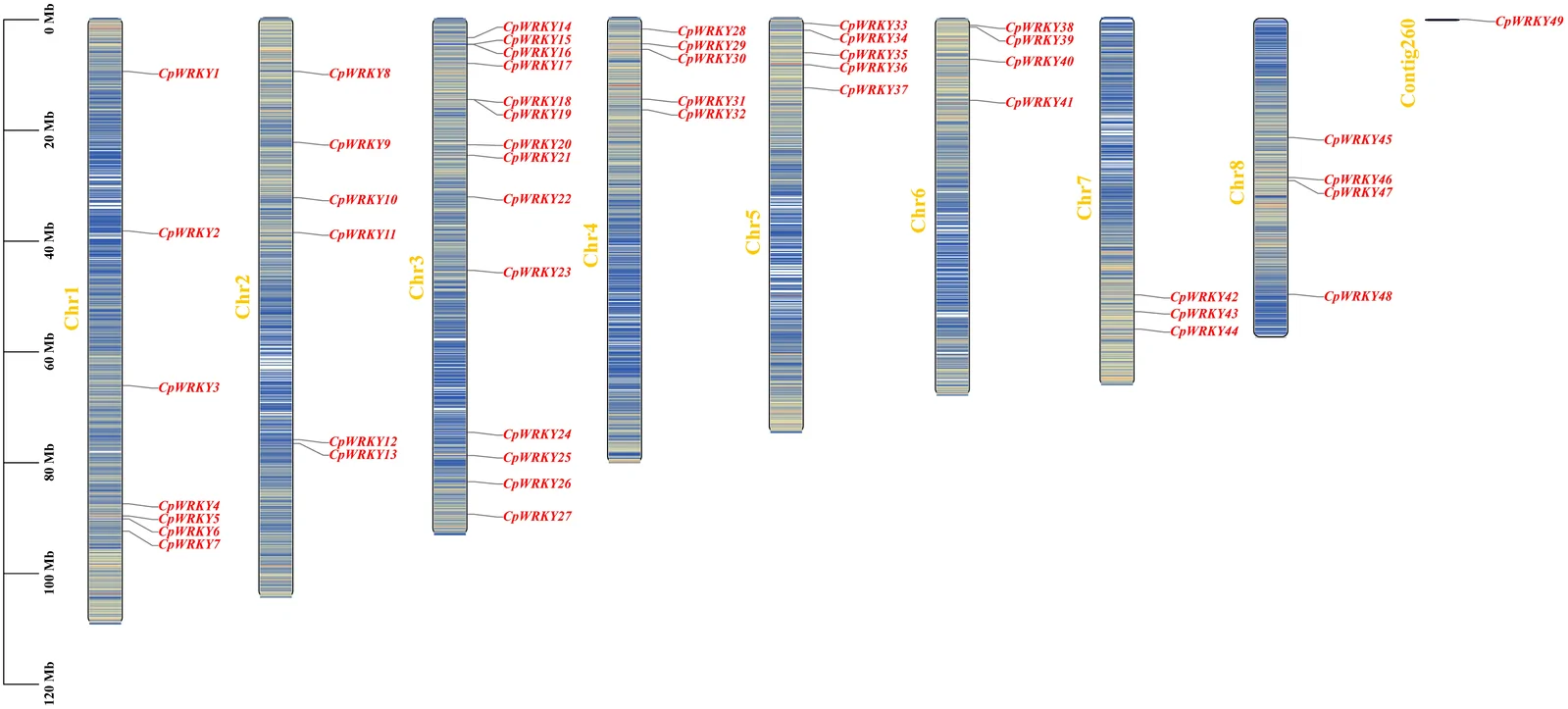
Chromosomal distribution of *CpWRKYs*. The left scale indicates the lengths (Mb) of the eight chromosomes. Gray lines mark the precise positions of individual genes on their respective chromosomes, and the color gradient along the chromosomes represents gene density.

**Table 1:** Basic information and physicochemical properties of the CpWRKY TF family members.

Gene name	Gene ID	AA	MW	pI	II	GRAVY	Subcellular localization
*CpWRKY1*	Cpil01G000603.t1	200	22678.83	9.01	45.03	-0.67	Chloroplast
*CpWRKY2*	Cpil01G001360.t1	269	30877.22	8.96	47.47	-0.64	Nuclear
*CpWRKY3*	Cpil01G002412.t1	338	37275.23	6.46	62.11	-0.908	Nuclear
*CpWRKY4*	Cpil01G003461.t1	185	21391.08	9.38	50.05	-0.975	Nuclear
*CpWRKY5*	Cpil01G003605.t1	294	32284.68	6.4	47.11	-0.744	Nuclear
*CpWRKY6*	Cpil01G003648.t1	550	60117.42	7.72	60.63	-0.863	Nuclear
*CpWRKY7*	Cpil01G003768.t1	557	61175.93	8.63	49.13	-0.772	Nuclear
*CpWRKY8*	Cpil02G000652.t1	346	38086.26	8.47	45.92	-0.809	Nuclear
*CpWRKY9*	Cpil02G001410.t1	363	40044.05	9.7	57.17	-0.729	Nuclear
*CpWRKY10*	Cpil02G002025.t1	577	62433.04	8.42	56.14	-0.764	Nuclear
*CpWRKY11*	Cpil02G002360.t1	165	18887.15	8.46	53.79	-0.929	Nuclear
*CpWRKY12*	Cpil02G003396.t1	194	22158.43	6.23	54.76	-0.887	Nuclear
*CpWRKY13*	Cpil02G003416.t1	103	12435.14	9.62	33.76	-1.179	Nuclear
*CpWRKY14*	Cpil03G000183.t1	361	40950.25	5.4	57.32	-0.792	Nuclear
*CpWRKY15*	Cpil03G000255.t1	208	23328.34	9.21	59.57	-0.697	Peroxisome
*CpWRKY16*	Cpil03G000257.t1	334	37305.55	5.11	56.76	-0.663	Nuclear
*CpWRKY17*	Cpil03G000462.t1	318	34608.7	6.99	49.83	-0.574	Nuclear
*CpWRKY18*	Cpil03G000909.t1	322	34773.64	5.45	47.88	-0.589	Nuclear
*CpWRKY19*	Cpil03G000910.t1	326	36938.27	5.38	51.01	-0.722	Nuclear
*CpWRKY20*	Cpil03G001396.t1	528	57836.96	7.22	51.54	-0.83	Nuclear
*CpWRKY21*	Cpil03G001498.t1	518	56817.24	8.57	59.19	-0.84	Nuclear
*CpWRKY22*	Cpil03G001920.t1	322	35216.01	9.59	49.66	-0.525	Nuclear
*CpWRKY23*	Cpil03G002601.t1	580	62798.68	7.79	46.93	-0.647	Nuclear
*CpWRKY24*	Cpil03G003388.t1	510	55757.61	5.56	51.34	-0.742	Nuclear
*CpWRKY25*	Cpil03G003524.t1	316	35369.33	7.31	57.84	-0.88	Nuclear
*CpWRKY26*	Cpil03G003696.t1	293	32491.62	5.51	48.83	-0.849	Nuclear
*CpWRKY27*	Cpil03G004033.t1	346	38326.33	6.06	55.34	-0.785	Nuclear
*CpWRKY28*	Cpil04G000115.t1	728	79133.4	6.23	53.02	-0.751	Nuclear
*CpWRKY29*	Cpil04G000306.t1	604	65742.72	6.62	49.93	-0.735	Nuclear
*CpWRKY30*	Cpil04G000384.t1	986	107946.28	6.25	40.12	-0.312	Nuclear
*CpWRKY31*	Cpil04G001015.t1	516	56995.01	6.61	46.69	-0.931	Nuclear
*CpWRKY32*	Cpil04G001131.t1	615	66652.31	6.24	44.65	-0.767	Nuclear
*CpWRKY33*	Cpil05G000065.t1	313	35389.43	5.9	63.23	-0.682	Nuclear
*CpWRKY34*	Cpil05G000134.t1	514	57242.43	6.88	57.03	-1.07	Nuclear
*CpWRKY35*	Cpil05G000428.t1	288	32444.18	4.91	55.37	-0.622	Nuclear
*CpWRKY36*	Cpil05G000600.t1	439	48893.61	8.87	41.24	-0.835	Nuclear
*CpWRKY37*	Cpil05G000892.t1	511	56321.18	5.77	54.15	-0.775	Nuclear
*CpWRKY38*	Cpil06G000093.t1	346	38127.59	5.99	44.55	-0.618	Nuclear
*CpWRKY39*	Cpil06G000118.t1	267	30436.55	4.87	75.09	-1.005	Nuclear
*CpWRKY40*	Cpil06G000545.t1	345	38614.66	9.55	63.56	-0.817	Nuclear
*CpWRKY41*	Cpil06G001050.t1	590	64912.28	8.34	72.32	-0.915	Nuclear
*CpWRKY42*	Cpil07G001691.t1	168	19058.87	5.45	36.17	-0.961	Cytoplasm
*CpWRKY43*	Cpil07G001894.t1	269	29380.12	9.99	54.44	-0.662	Nuclear
*CpWRKY44*	Cpil07G002138.t1	512	55665.91	5.84	53.11	-0.715	Nuclear
*CpWRKY45*	Cpil08G000841.t1	290	34046.17	9.67	58.81	-1.054	Nuclear
*CpWRKY46*	Cpil08G001298.t1	342	37191.98	6	66.5	-0.815	Nuclear
*CpWRKY47*	Cpil08G001345.t1	336	37972.54	5.38	46.68	-0.653	Nuclear
*CpWRKY48*	Cpil08G002579.t1	734	79569.73	5.58	47.55	-0.552	Nuclear
*CpWRKY49*	Cpil260G000001.t1	200	22797.92	8.88	38.6	-0.709	Nuclear

AA, number of amino acids; MW, molecular weight; pI, isoelectric point; GRAVY, grand average of hydropathicity; II, instability index.

### 3.2 Phylogenetic analysis of CpWRKYs

To determine the phylogenetic relationships of the *CpWRKY* gene family, an ML phylogenetic tree was constructed using TBtools based on the WRKY protein sequences from *C. pilosula* and the model plant *A. thaliana*. Based on their clustering patterns, protein sequence similarity, and previously established classification criteria, the 49 CpWRKYs were divided into seven subfamilies: Group I (10 members), IIa (2), IIb (7), IIc (14), IId (4), IIe (6), and Group III (6) (**Figure 2**).

**Figure 2:**
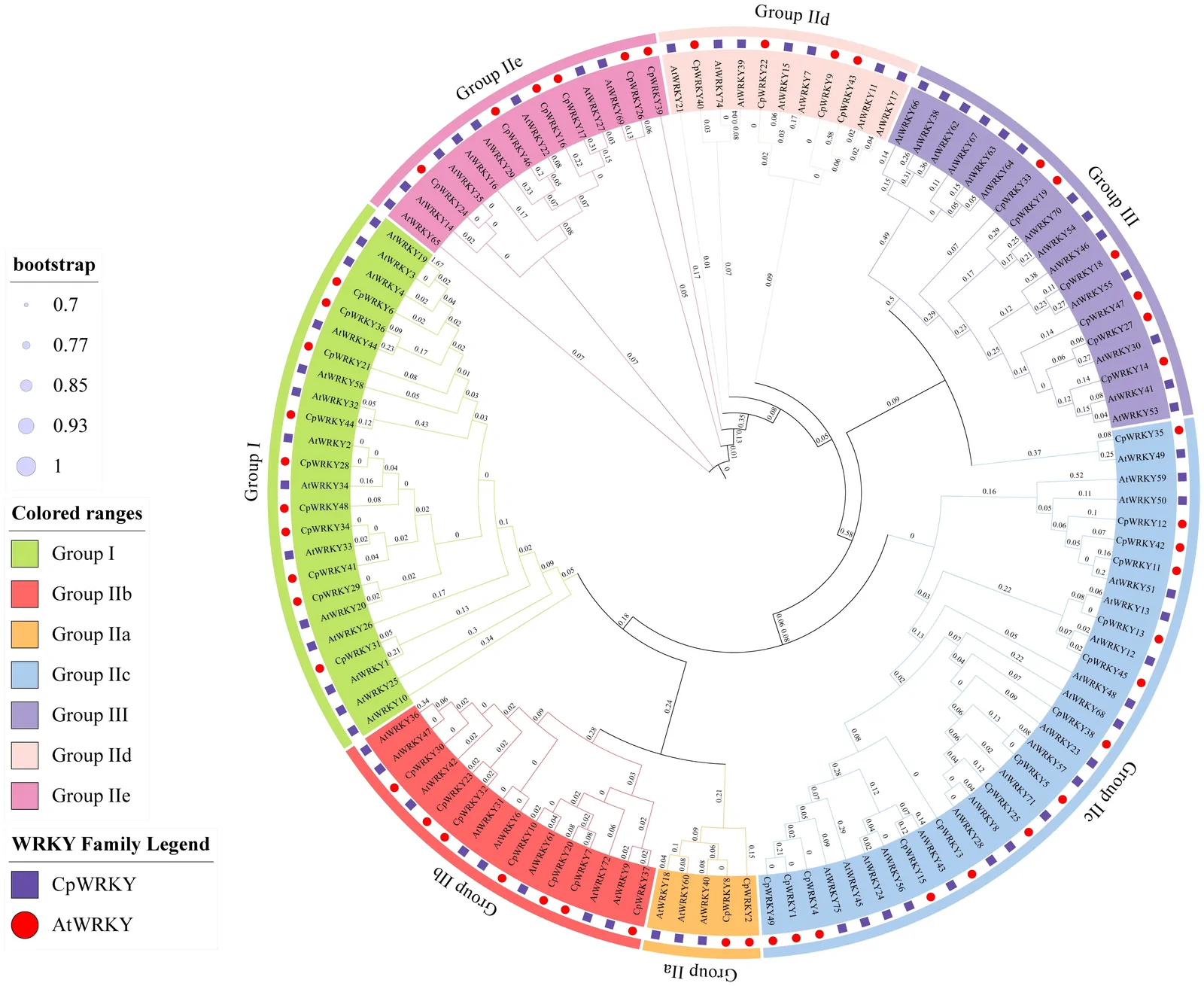
Phylogenetic tree of the WRKY gene family in *C. pilosula* and *A. thaliana*. AtWRKYs are shown as red circles, and CpWRKYs as purple squares.

### 3.3 Conserved motif and gene structure analyses

To explore the evolutionary and functional relationships within the *CpWRKY* gene family, we performed a joint analysis of phylogeny, conserved domains, and motif predictions. Based on evolutionary relationships and sequence similarity, the proteins were classified into seven subfamilies (**Figure 3A**). To further elucidate the potential functions of CpWRKYs, we analyzed conserved motifs and gene structures using the MEME website with the number of motifs set to 10. Sequence analysis revealed that the distribution of these motifs exhibited significant subfamily-specific patterns (**Figures 3B, C**), which verified the reliability of our gene identification. In particular, Motif 1 was present in all 49 CpWRKY members and contains the highly conserved WRKYGQK heptapeptide, which is the hallmark feature defining the WRKY family (**Figure 3D**).

**Figure 3:**
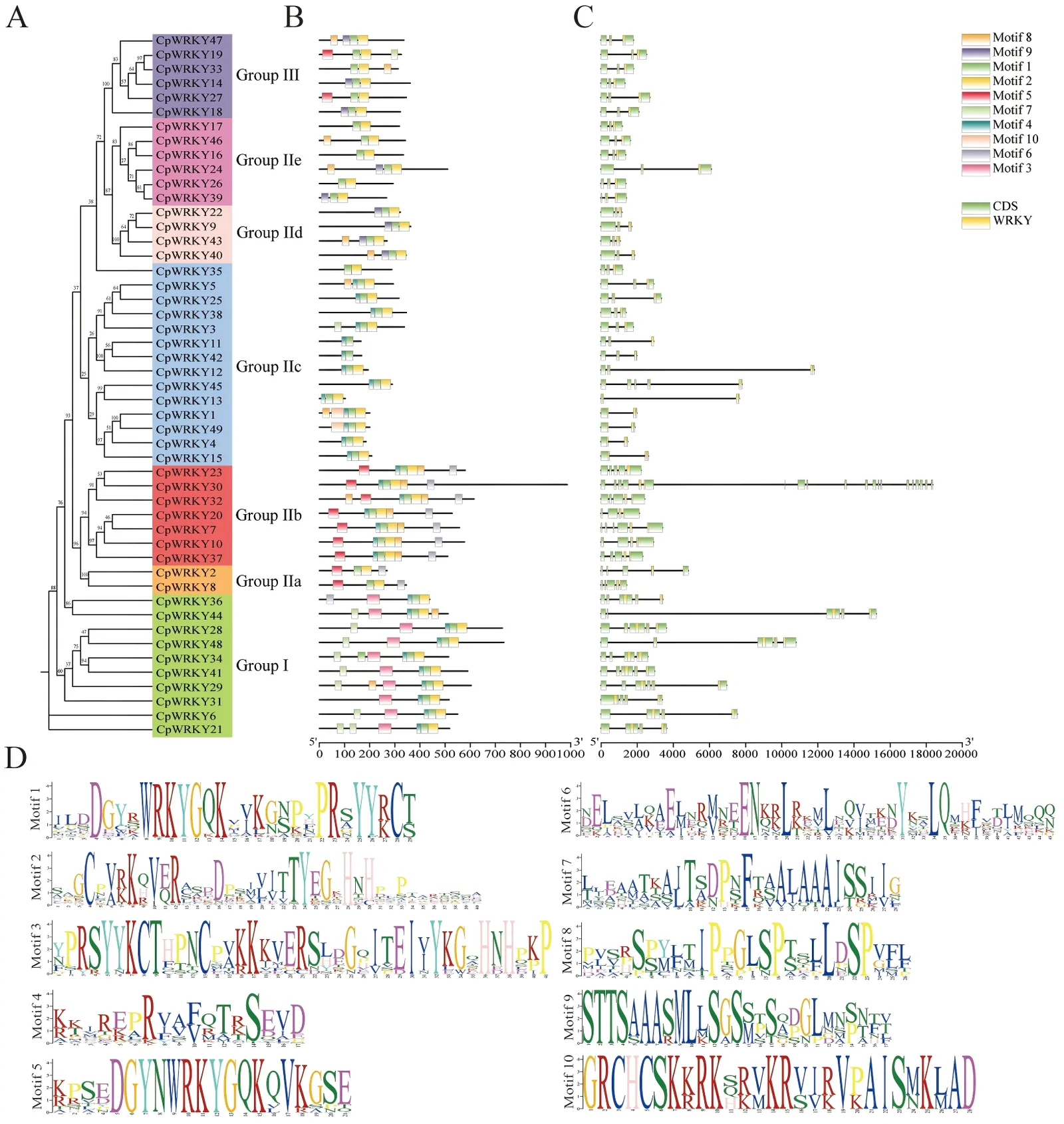
Conserved motifs and gene structures of WRKYs in *C. pilosula*. **(A)** Phylogenetic tree of CpWRKYs, with different subfamilies indicated by colored blocks. **(B)** Motif analysis of CpWRKYs; conserved motifs are shown as colored boxes, and non-conserved sequences as black lines. **(C)** Gene structure organization of CpWRKYs, with coding regions in green and WRKY domains in yellow. **(D)** Sequence logos of conserved motifs in CpWRKY proteins. Scale bar represents gene length in kilobases (kb).

Except for CpWRKY11 and CpWRKY42, all other members contained Motif 2. The distribution patterns of various motifs across different subfamilies further supported the reliability of the phylogenetic classification. Group I was the most distinctive, with all its members uniquely possessing Motif 3 and most containing Motif 7. Motif 4 was widespread in Groups I, IIb, and IIc (except CpWRKY35). Motif 5 was mainly found in all members of Groups IIa and IIb, as well as in CpWRKY27 and CpWRKY19 of Group III. Motif 6 was restricted to Groups I, IIa, and IIb. Motif 9 was specifically distributed in Groups IId, IIe, and III. In contrast, Motifs 8 and 10 were scattered across subfamilies without clear patterns. Overall, members within the same subfamily shared highly similar conserved motif compositions, providing a structural basis for the performance of analogous biological functions.

### 3.4 Promoter cis-acting element and synteny analyses

To further elucidate the potential regulatory roles of *CpWRKYs* in plant biological processes, we analyzed the 2 000 bp promoter regions upstream of all *CpWRKYs* and ordered the results according to the phylogenetic tree (**Figure 4A**). Promoter cis-element analysis identified 19 types of CREs, which were classified into three major categories: stress response, plant development, and phytohormone response. All members contained light-responsive elements and hormone-responsive elements; only *CpWRKY43* lacked stress-responsive elements (low temperature, drought, oxygen deprivation, etc.). Among these, MeJA-responsive (158) and ABA-responsive (102) elements were the most abundant (**Figure 4B**). Several members, such as *CpWRKY16* and *CpWRKY35*, contained multiple stress-related elements, suggesting their potential involvement in cross-talk regulation among various stresses.

**Figure 4:**
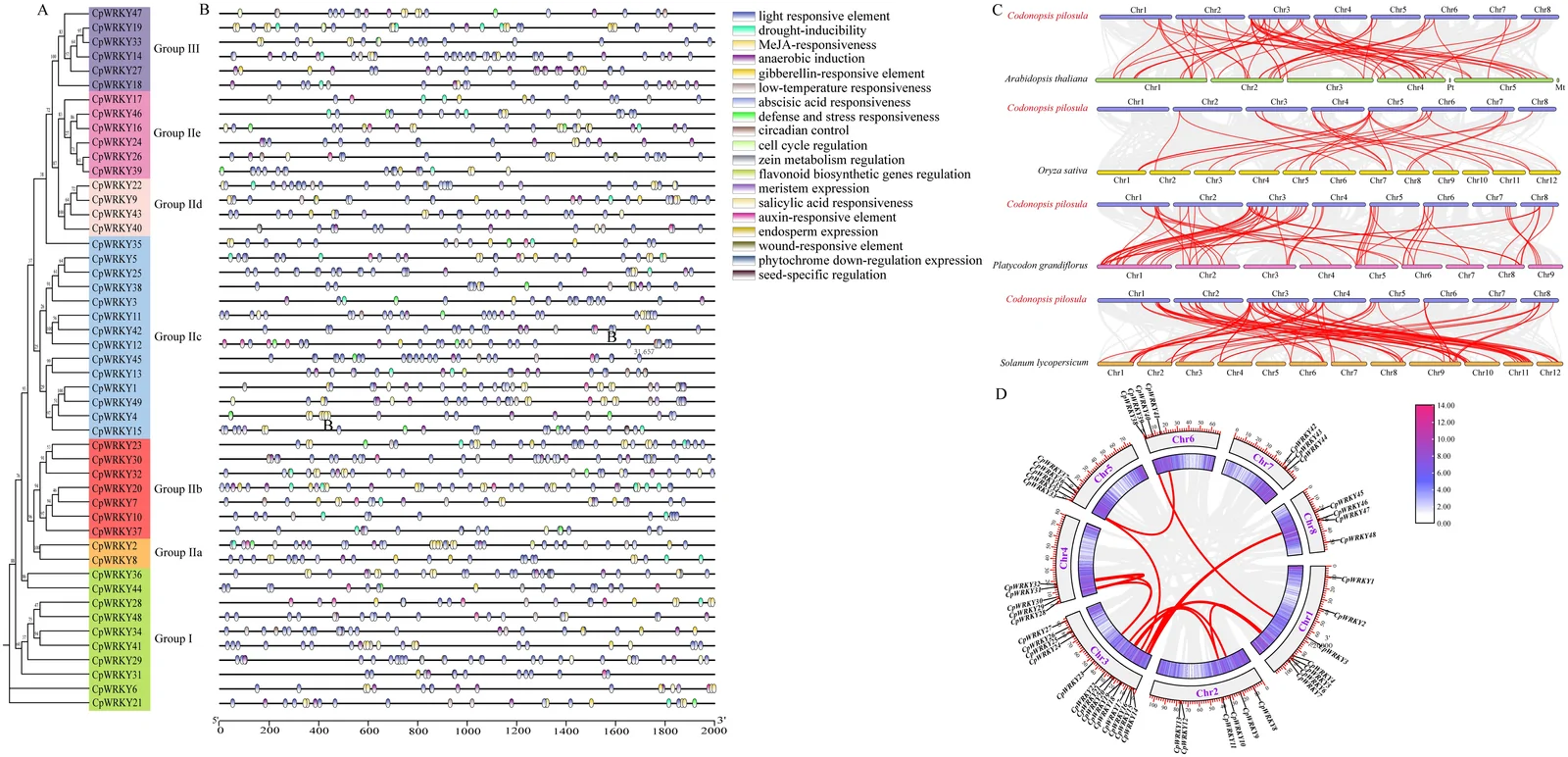
Distribution of cis-acting elements and synteny analysis of *CpWRKYs*. **(A)** Phylogenetic tree of CpWRKYs, with subfamilies color-coded. **(B)** Distribution of CREs in *CpWRKY* promoters; different colored boxes represent different element types. **(C)** Synteny relationships between *C. pilosula* and *A. thaliana*, *O. sativa*, *P. grandiflorus*, and *S. lycopersicum*. Gray lines in the background indicate syntenic regions between genomes, and red lines indicate syntenic WRKY gene pairs. **(D)** Chromosomal distribution and gene duplication relationships of *CpWRKYs*. Gray lines represent syntenic regions within the *C. pilosula* genome, and red lines indicate duplicated *CpWRKY* gene pairs.

Synteny analysis revealed that CpWRKY family members shared the highest number of syntenic gene pairs with the dicot *S. lycopersicum* (75 pairs), followed by the same-family species *P. grandiflorus* (62 pairs) and *A. thaliana* (59 pairs), whereas the fewest were observed with the monocot *O. sativa* (26 pairs) (**Figure 4C**). Intraspecific synteny analysis identified 16 pairs of duplicated genes, all of which occurred within the same subfamily. Group II contributed the largest proportion. Most of the duplicated gene pairs (13/16) were located on different chromosomes, suggesting that interchromosomal segmental duplication may have contributed substantially to the expansion of the *CpWRKY* gene family (**Figure 4D**).

### 3.5 Bioinformatic characterization of *CpWRKY8*

Bioinformatic analysis showed that CpWRKY8 comprised 346 amino acids (**Supplementary Table S2**), with its secondary structure predominantly composed of α-helices (29.77%) and random coils (57.51%). Its tertiary structure was predicted by homology modeling using SWISS-MODEL, with the Actinidia chinensis WRKY DNA-binding protein 40 (A0A7J0GAW0.1.A) as the template. Sequence alignment revealed 71.29% identity and 0.92 coverage between CpWRKY8 and the template, and the model received a GMQE score of 0.63, indicating moderate confidence in the predicted model. (**Supplementary Figure S1**). This protein lacks transmembrane domains and a typical secretory signal peptide, while its full sequence is predicted to contain multiple phosphorylation sites and three potential N-glycosylation sites, located at asparagine residues at positions 113, 286 and 293, respectively. Phylogenetically, CpWRKY8 clustered most closely with GmWRKY40 from *Glycine max*, supporting its classification as a Group IIa member.

### 3.6 *CpWRKY8* is localized to the nucleus and possesses transcriptional activation activity

Subcellular localization assays showed that the green fluorescence of the 35S::EGFP-CpWRKY8 fusion protein co-localized exclusively with the nuclear dye DAPI, whereas the empty vector EGFP fluorescence was distributed throughout the cell (**Figure 5A**), confirming that CpWRKY8 is a nuclear-localized protein.

**Figure 5:**
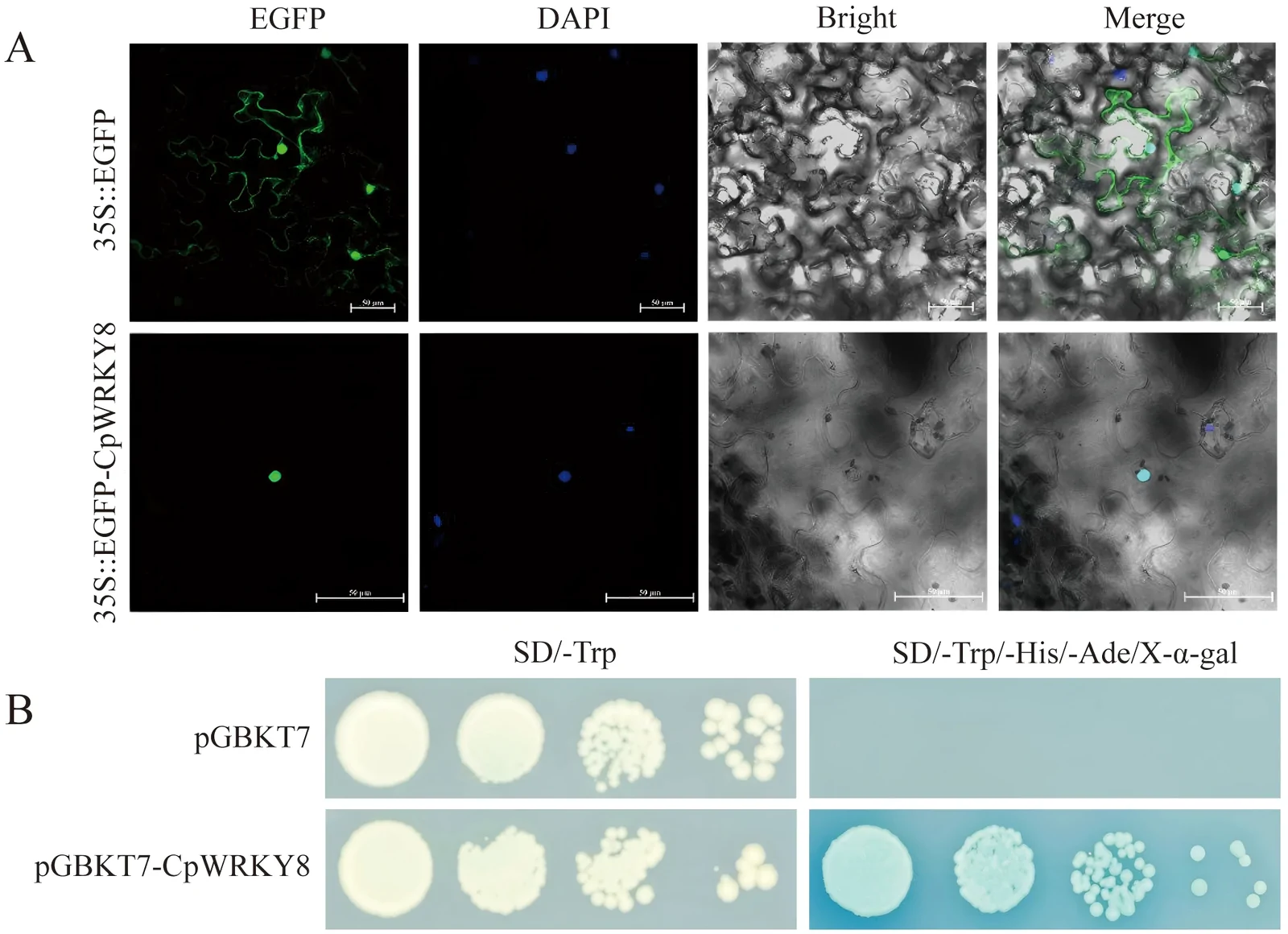
Subcellular localization and transcriptional activation activity of *CpWRKY8*. **(A)** Subcellular localization of *CpWRKY8* in *N. benthamiana* epidermal cells. 35S::EGFP was used as the empty vector control. Confocal images show EGFP (green), DAPI nuclear staining (blue), bright field, and merged images. Scale bar = 50 µm. **(B)** Transcriptional activation assay of *CpWRKY8*, with pGBKT7 as the empty vector control.

Assays of yeast transcriptional activity showed that yeast transformed with the plasmid pGBKT7-CpWRKY8, which contains a conserved fragment of the *CpWRKY8* core CDS, grew normally on SD/-Trp/-His/-Ade/X-α-gal medium and turned blue, whereas the empty vector failed to support growth (**Figure 5B**), demonstrating that *CpWRKY8* possesses transcriptional activation activity.

### 3.7 Overexpression of *CpWRKY8* enhances drought tolerance in *N. tabacum* by augmenting antioxidant capacity

Through leaf disc transformation and hygromycin selection, a total of eight independent transgenic lines were obtained. Genomic PCR confirmed the presence of the target band in all lines (**Figure 6A**). The qRT-PCR results revealed that three lines (OE5, OE6, and OE7) had significantly higher expression levels than the WT (**Figure 6B**) and were therefore selected for subsequent experiments.

**Figure 6:**
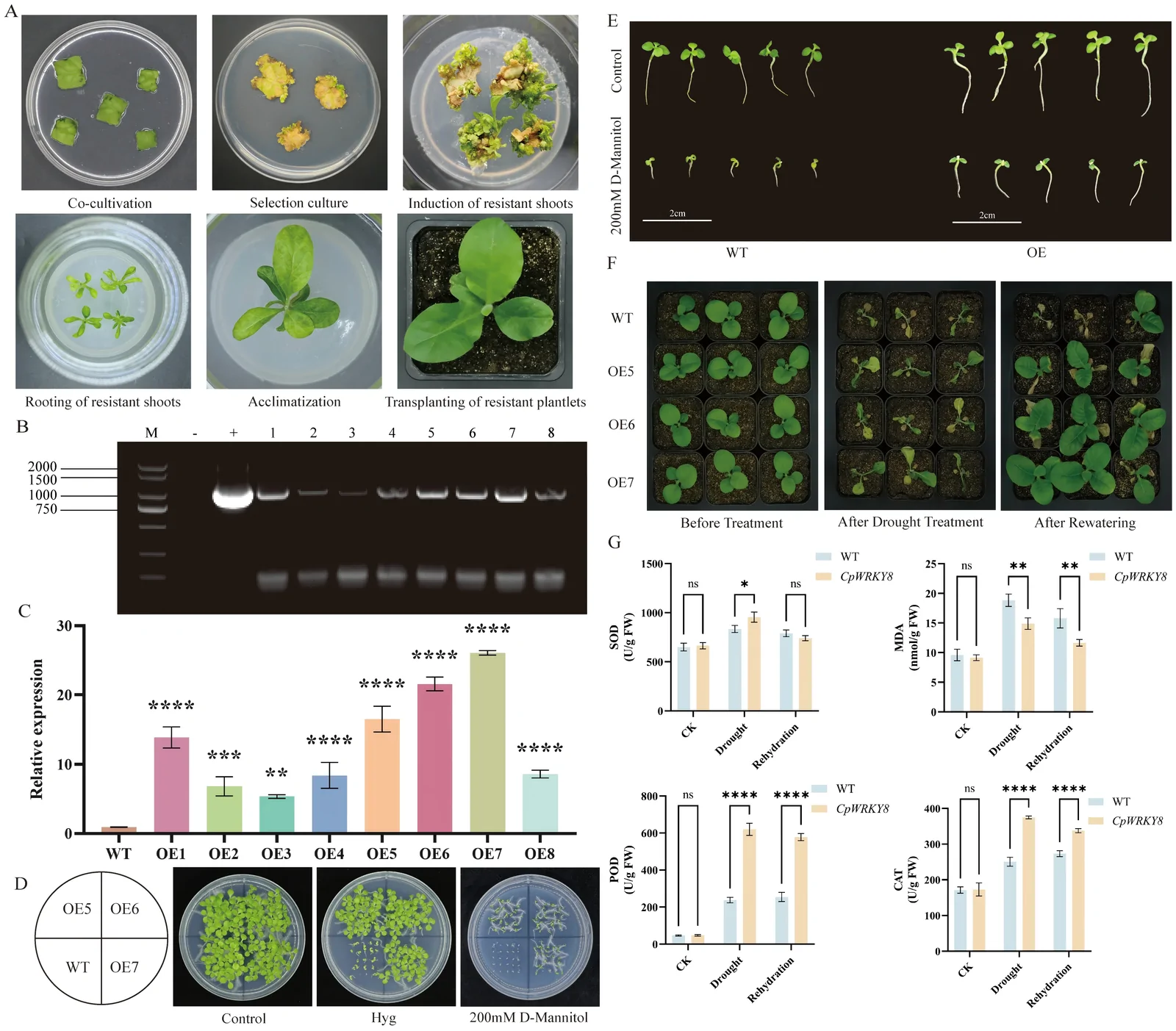
Overexpression of *CpWRKY8* enhances drought tolerance and antioxidant capacity in transgenic *N. tabacum*. **(A)** Genetic transformation process of transgenic tobacco: co-cultivation, selection, resistant shoot induction, rooting, rooting culture, and acclimatization. **(B)** PCR identification of transgenic tobacco. M, DL2 000 DNA marker; lanes 1-8, independent transgenic lines; +, positive control (fusion expression vector plasmid); WT, wild-type; -, negative control (ddH_2_O). **(C)** Relative expression levels of *CpWRKY8* in different transgenic lines. **P<0.01, ***P<0.001. **(D)** Seed screening and stress tolerance assay of transgenic tobacco. **(E)** Post-germination growth phenotypes under osmotic stress. **(F)** Phenotypes of transgenic tobacco under drought stress and after rehydration. **(G)** Physiological and biochemical indices of transgenic tobacco under drought stress. Data are presented as mean ± SD of three independent experiments (one-way ANOVA followed by Tukey’s HSD test; *P < 0.05; **P < 0.01; ns, not significant).

On standard medium, the germination rates of both WT and *CpWRKY8* OE *N. tabacum* seeds were close to 100%. On medium containing Hyg, *CpWRKY8* OE seeds germinated and grew normally, whereas WT seedlings turned yellow and died after germination. On stress medium containing 200 mM mannitol, OE seeds maintained a germination rate of approximately 96%, with longer roots and darker cotyledons. In contrast, the WT germination rate dropped to 92%, and seedling growth was severely inhibited (**Figure 6C**). After 14 days of natural drought stress, WT leaves exhibited severe wilting and chlorosis, whereas the OE lines displayed much milder symptoms. Following 7 days of rehydration, the OE lines recovered more rapidly and exhibited significantly higher survival rates than the WT (**Figure 6D**). Post-germination osmotic stress assays showed that, under 200 mM D-mannitol treatment, the OE lines exhibited significantly greater primary root length, hypocotyl length, true leaf number, and lateral root number than the WT (**Figure 6E**).

Under normal growth conditions, there were no significant differences in SOD, POD, and CAT activities or MDA content between the WT and OE lines. Following drought treatment, antioxidant enzyme activities increased in all plants. However, the OE lines showed significantly higher SOD, POD, and CAT activities, and significantly lower MDA accumulation, compared with the WT (**Figure 6F**). After rehydration, the physiological indices of the OE lines recovered to a greater extent than those of the WT. Collectively, these results indicate that *CpWRKY8* enhances drought tolerance, which is associated with increased antioxidant enzyme activities and reduced membrane lipid peroxidation.

### 3.8 CpPYR/PYL activates *CpWRKY8* promoter-driven reporter gene expression in heterologous systems

Y1H assays revealed that yeast co-transformed with pHIS2-*CpWRKY8* and pGADT7-CpPYR/PYL1–4 produced significantly more colonies on medium containing 115 mM 3-AT than the negative control (**Figure 7A**), indicating that CpPYR/PYL can bind to the *CpWRKY8* promoter in yeast.

**Figure 7:**
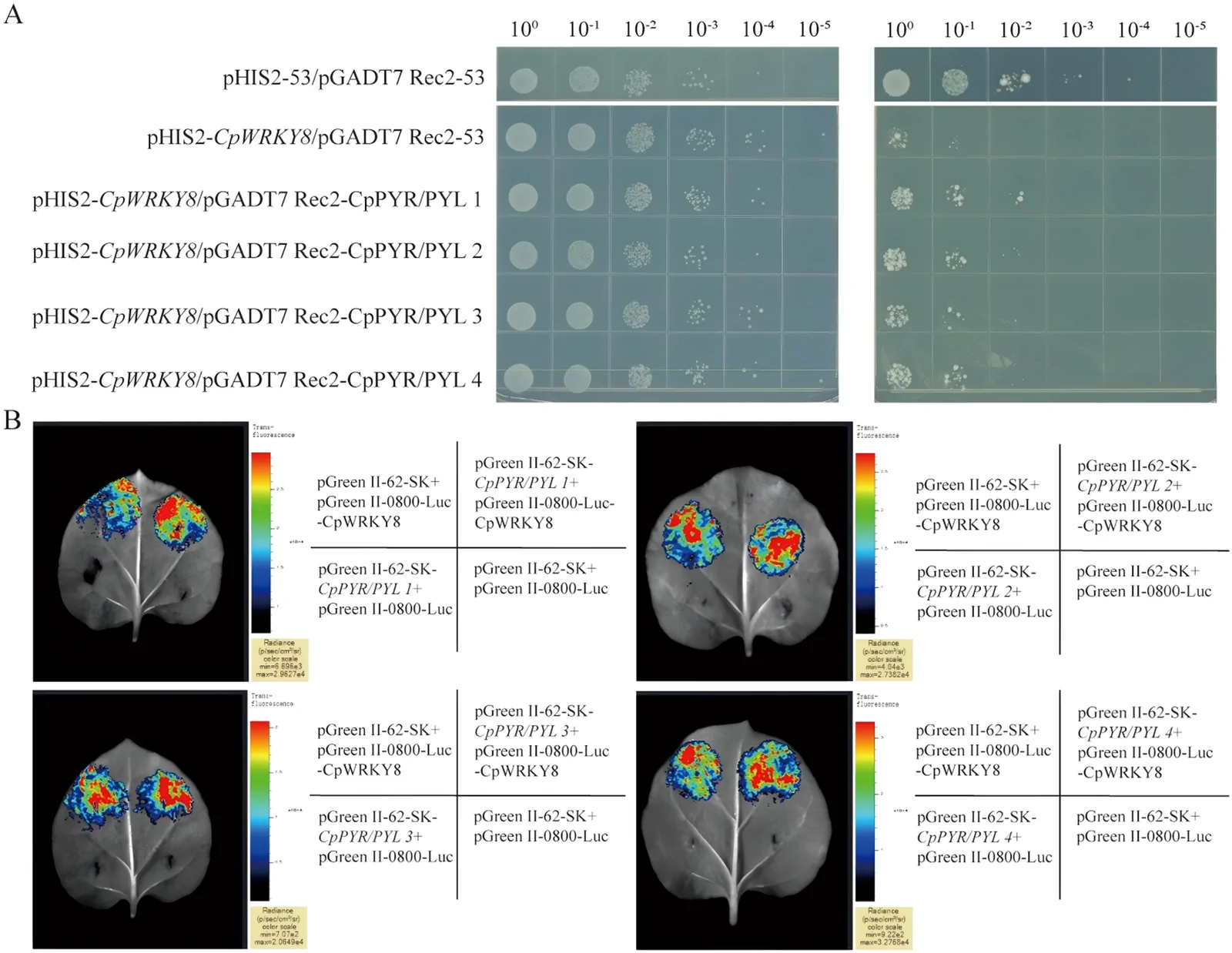
CpPYR/PYL activates *CpWRKY8* promoter-driven reporter gene expression in heterologous systems. **(A)** Y1H assay using pGADT7 Rec2-53-CpPYR/PYL and pHIS2-*CpWRKY8* shows activation of the HIS3 reporter. **(B)** DLR assay using pGreen II-62-SK-*CpPYR/PYL* and pGreen II-0800-Luc-CpWRKY8 shows enhanced LUC expression.

DLR assays further confirmed that co-expression of pGreen II-62-SK-*CpPYR/PYL*1–4 with the reporter pGreen II-0800-Luc-CpWRKY8 in *N. benthamiana* leaves resulted in significantly higher LUC/REN ratios than the control (**Figure 7B**), demonstrating that CpPYR/PYL positively regulates the transcriptional activity of the *CpWRKY8* promoter.

## 4 Discussion

This study presents the first genome-wide systematic identification of the WRKY TF family in the medicinal plant *C. pilosula* and, based on this, provides a thorough characterization of the biological function of *CpWRKY8* in drought signal response and its upstream regulatory mechanisms. We identified 49 WRKY members in the *C. pilosula* genome, a number that differs from those reported in *A. thaliana* (72), *O. sativa* (102), *P. grandiflorus* (27), and *Sophora flavescens* (69) (Eulgem et al., 2000; Wu et al., 2005; Li et al., 2022, Li et al., 2024). These differences may be attributable to species-specific evolutionary histories and gene duplication events (Cannon et al., 2004; Li et al., 2017, Li et al., 2026b). Phylogenetic and conserved motif analyses revealed that all CpWRKY members contain the highly conserved WRKYGQK domain and a typical zinc-finger motif. Their subfamily classification is largely consistent with that of *A. thaliana*, indicating significant evolutionary conservation of this family in dicotyledonous plants (Wang et al., 2011).

Functionally, *CpWRKY8* was localized to the nucleus and possessed transcriptional activation activity, consistent with findings for other Group IIa subfamily members such as *BnaWRKY40* and *TfWRKY40* (Sun et al., 2024; Xu et al., 2025), and in line with the fundamental characteristics of classical TFs (Calkhoven and Ab, 1996; Dhatterwal et al., 2024). Notably, the Group IIa subfamily has been shown to serve as a central hub integrating ABA signaling and drought responses in *A. thaliana*, with its members often fine-tuning the expression of downstream genes through synergistic or antagonistic actions (Grzechowiak et al., 2022; Wen, 2025). In our study, heterologous overexpression of *CpWRKY8* in *N. tabacum* significantly improved the survival rate and post-rehydration recovery of transgenic plants under drought stress. Physiological analyses indicated that this enhanced drought tolerance was closely associated with significantly increased SOD, POD, and CAT activities, and a marked reduction in MDA content. This finding is highly consistent with previous reports on other WRKY TFs, such as *MfWRKY40* in *M. falcata* and *GhWRKY6* in *G. hirsutum*, which mitigate oxidative damage by modulating the antioxidant enzyme system (Li et al., 2019; Zhang et al., 2025b). Collectively, these observations suggest that antioxidant-associated drought responses may represent a conserved functional feature of some Group IIa WRKY members, and that the observed activity of *CpWRKY8* in the heterologous *N. tabacum* system is consistent with this conserved role.

Having elucidated the downstream function of *CpWRKY8*, we further investigated its upstream regulatory mechanisms. Promoter cis-element analysis revealed that the *CpWRKY8* promoter region is enriched with multiple ABA-responsive elements (**Figure 4B**), suggesting that its transcriptional activity may be regulated by downstream TFs of the ABA signaling pathway. In the canonical ABA signaling pathway, PYR/PYL receptors activate SnRK2 kinases by inhibiting PP2C phosphatase activity. The activated SnRK2 then phosphorylates and activates downstream TFs such as AREB/ABF, which initiate the expression of a suite of stress-responsive genes (Fujii et al., 2009). However, CpPYR/PYL proteins belong to a class of ligand-binding proteins containing a START domain and, as such, lack intrinsic DNA-binding capacity. Therefore, the observed activation of the *CpWRKY8* promoter by CpPYR/PYL in our Y1H and DLR assays likely resulted from indirect regulatory mechanisms in the heterologous systems. In the yeast system, CpPYR/PYL may indirectly activate the HIS3 reporter gene through protein-protein interactions with endogenous yeast TFs (Ouwerkerk and Meijer, 2001). In the *N. benthamiana* transient expression system, CpPYR/PYL may activate the endogenous PP2C-SnRK2-AREB/ABF signaling cascade, which in turn indirectly enhances *CpWRKY8* promoter activity (Dejonghe et al., 2018; Li et al., 2023a). This inference is supported by the presence of ABREs in the *CpWRKY8* promoter, as ABREs are the core recognition and binding sites for AREB/ABF TFs (Dejonghe et al., 2018; Li et al., 2023a). That said, given the heterologous nature of these assays, it is also possible that CpPYR/PYL modulates other intermediate TFs present in *N. benthamiana*, or influences promoter activity through alternative protein–protein interaction networks independent of the canonical PP2C-SnRK2 pathway.

Based on these findings, we propose a working model of an ABA-CpPYR/PYL signaling cascade-*CpWRKY8* transcriptional response (**Figure 8**). In this model, drought stress leads to increased ABA levels, which activate the CpPYR/PYL-PP2C-SnRK2 cascade, indirectly upregulating *CpWRKY8* expression via downstream AREB/ABF TFs, ultimately enhancing antioxidant enzyme activities and improving drought tolerance. The dashed lines and arrows in **Figure 8** indicate the steps that are inferred based on the canonical ABA signaling pathway and the ABRE enrichment in the *CpWRKY8* promoter, rather than directly demonstrated in this study.

**Figure 8:**
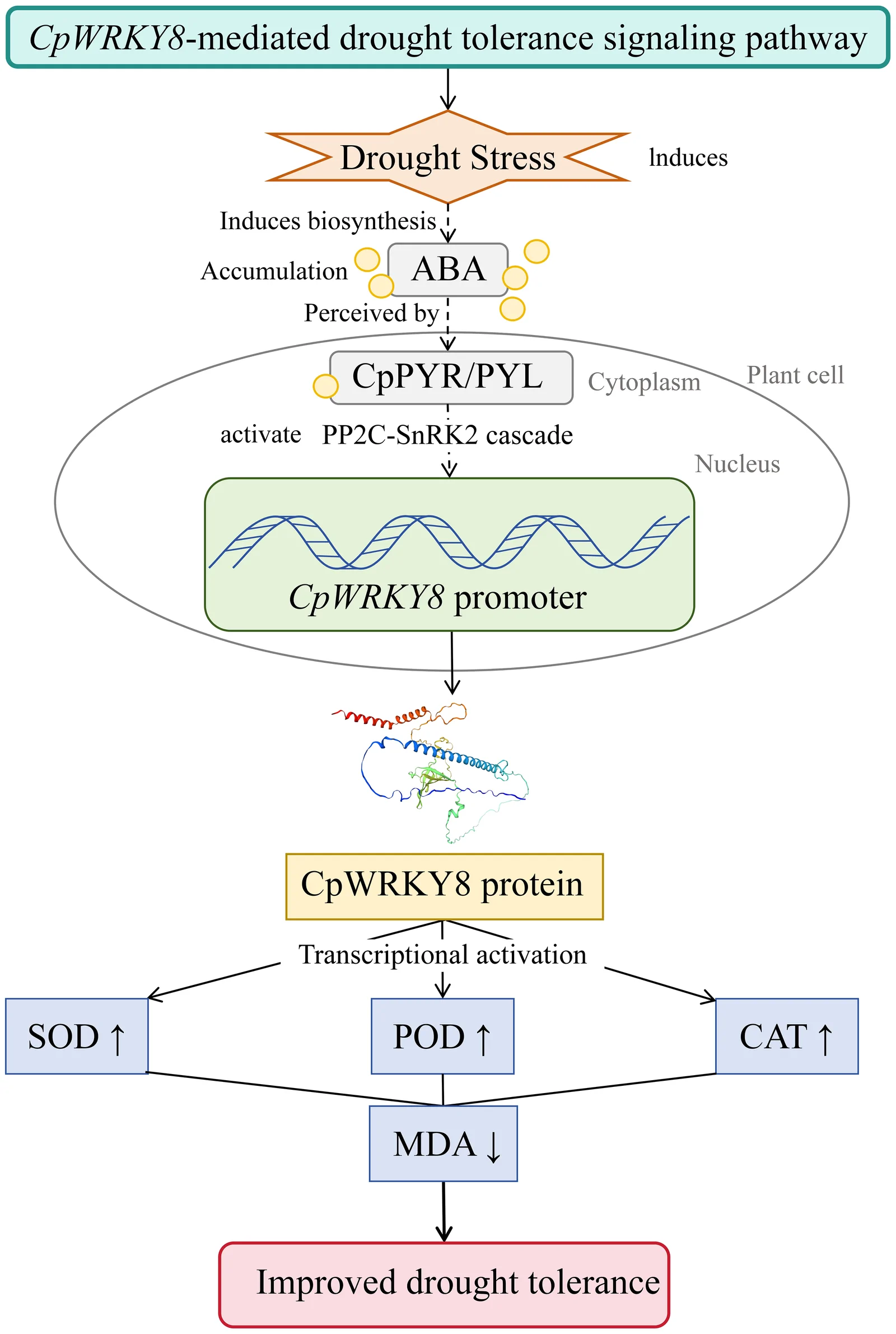
Proposed model illustrating the mechanism of *CpWRKY8* in response to drought stress.

Drought stress has been reported to affect the accumulation of bioactive compounds in *C. pilosula*, including lobetyolin and polysaccharides (Liang et al., 2021; Wang et al., 2024, Wang et al., 2025, Wang et al., 2026a). In parallel, WRKY TFs have been implicated in the regulation of specialized metabolism in several medicinal plants. For instance, in *Salvia miltiorrhiza*, *SmWRKY70* positively regulates tanshinone accumulation while simultaneously enhancing drought adaptability (Yu et al., 2026). In *Scutellaria baicalensis*, the drought-responsive *SbWRKY34* is involved in flavonoid metabolism (Zhang et al., 2025a). In *Isatis indigotica*, the drought responsive IiWRKY34 positively regulates lignan biosynthesis (Xiao et al., 2020). Together with our finding that *CpWRKY8* enhances drought tolerance, these observations raise the possibility that *CpWRKY8* may also participate in the regulation of drought-associated secondary metabolism in *C. pilosula*. These findings provide a foundation for future investigations into the relationship between drought tolerance and quality formation in *C. pilosula*.

In summary, this study not only provides foundational data for a systematic understanding of the *CpWRKY* gene family but also reveals the positive regulatory role of *CpWRKY8* in drought responses and its upstream ABA signaling cascade. This finding provides new insights into the molecular basis of drought tolerance in *C. pilosula* and supports the concept of “stress breeds quality” by highlighting a potential molecular connection between stress adaptation and medicinal quality formation. However, the direct contribution of *CpWRKY8* to the accumulation of pharmacologically active constituents remains to be determined. The identified *CpWRKY8* gene provides a potential genetic resource and a theoretical basis for molecular breeding aimed at improving drought tolerance in *C. pilosula* for cultivation in arid and semi-arid regions.

## 5 Summary

In this study, a total of 49 *CpWRKY* gene family members were identified, distributed across eight chromosomes and Contig260. All were hydrophilic proteins and predominantly localized to the nucleus. This study provides the first functional analysis of the *CpWRKY8* TF. We have confirmed that CpWRKY8 is a nuclear-localized transcriptional activator that, when overexpressed in *N. tabacum*, positively regulates drought tolerance by enhancing antioxidant enzyme activities and mitigating oxidative damage. Crucially, we found that the ABA receptor CpPYR/PYL positively regulates the transcription of *CpWRKY8* by indirectly enhancing *CpWRKY8* promoter activity. This discovery reveals a new dimension of crosstalk between ABA perception and WRKY-mediated stress responses via signaling cascades, broadening our understanding of the diverse mechanisms of ABA signaling and providing a theoretical basis and genetic resources for molecular breeding of drought-tolerant medicinal plants.

## Data Availability

The original contributions presented in the study are included in the article/**Supplementary Material**. Further inquiries can be directed to the corresponding authors.
