# Supplementary material for: Genome-wide identification of the WRKY gene family in Codonopsis pilosula and functional analysis of CpWRKY8 under drought stress
Source: Front Plant Sci. 2026 Sep 30;17:1961042. doi: 10.3389/fpls.2026.1961042 (PMC13661422; doi:10.3389/fpls.2026.1961042)
Supplement: Supplementary file 1 [file Supplementaryfile1.docx]

Supplementary Material

# 1 Supplementary Figures and Tables

For more information on Supplementary Material and for details on the different file types accepted, please see [here](https://www.frontiersin.org/guidelines/author-guidelines#supplementary-material).

## 1.1 Supplementary Figures

**Supplementary Figure 1.** Bioinformatic analysis of the *CpWRKY8* gene. (A) Nucleotide and deduced amino acid sequences of *CpWRKY8*. The asterisk (*) indicates the stop codon. (B) Secondary structure of the CpWRKY8 protein. (C) Tertiary structure of the CpWRKY8 protein. (D) Transmembrane structure of the CpWRKY8 protein. (E) Signal peptide prediction of the CpWRKY8 protein. (F) Phosphorylation site prediction of the CpWRKY8 protein. (G) Prediction of N-glycosylation sites in the CpWRKY8 protein. The purple line indicates the threshold, and the green line indicates the potential sites. (H) Phylogenetic analysis of CpWRKY8 and WRKY40 protein sequences from different species.

**Supplementary Figure 2.** Sanger sequencing validation of the cloned constructs. (A) Sequence alignment of the EGFP-CpWRKY8 construct. (B) Sequence alignment of the pGBKT7-CpWRKY8 construct. (C) Sequence alignment of the pHIS2-*CpWRKY8* construct. (D) Sequence alignment of the pGreenII 0800-LUC-CpWRKY8 construct. For each panel, the sequences are arranged as the forward homologous arm (Homologous arm-F), the reference sequence (the CpWRKY8 CDS for A and B; the CpWRKY8 promoter region (~2,000 bp) for C and D), the reverse homologous arm (Homologous arm-R), and the Sanger sequencing result.

## 1.2 Supplementary Table

**Supplementary Table 1.** Primers used in the experiment.

| **引物名称** | **引物序列（5'-3'）** |
| --- | --- |
| EGFP-*CpWRKY8*-F | agaacacgggggacgagctcATGGAATATACAAGCTTGGTTGATACTTCAT |
| EGFP-*CpWRKY8*-R | gccctctagaggatccCCATTTTTGGGTGTGATTAGACTGAAGA |
| pGBKT7-*CpWRKY8*-F | catggaggccgaattcATGGAATATACAAGCTTGGTTGATACTTCAT |
| pGBKT7-*CpWRKY8*-R | gcaggtcgacggatccCCATTTTTGGGTGTGATTAGACTGAAGA |
| Ntactin-F | GGTAGCTCCACCTGAGAGGAAGT |
| Ntactin-R | GCCTTTGCAATCCACATCTGT |
| pIHS2-CpWRKY18-F | tcactatagggcgaattccATGGAATATACAAGCTTGGTTGATACTTCAT |
| pIHS2-*CpWRKY18*-R | tcgcgaacgcgtgagctcCCATTTTTGGGTGTGATTAGACTGAAGA |
| pGADT7 Rec2-PYR/PYL1-F | gcagagtggccattatggcccgggATGAGCGGCAATGGATTTGG |
| pGADT7 Rec2-PYR/PYL1-R | gcggccgacatgttttttcccgggGATCCTATCAATGGGCTCAGTC |
| pGADT7 Rec2-PYR/PYL2-F | gcagagtggccattatggcccgggATGCCTTCAATCCAGTCATCAATT |
| pGADT7 Rec2-PYR/PYL2-R | gcggccgacatgttttttcccgggCTTCGTTTTGGCGGCCAAAT |
| pGADT7 Rec2-PYR/PYL3-F | gcagagtggccattatggcccgggATGCTTTCTAATCCTCCAAATTCCT |
| pGADT7 Rec2-PYR/PYL3-R | gcggccgacatgttttttcccgggGTTTCGACGGGGCAAATTCT |
| pGADT7 Rec2-PYR/PYL4-F | gcagagtggccattatggcccgggATGGGAAACAAGAAATGGTGTGG |
| pGADT7 Rec2-PYR/PYL4-R | gcggccgacatgttttttcccgggCTTAAAGTTTGTGGACGAAGCTTC |
| 0800-WRKY8-F | cggtatcgataagcttCTAATTGTCTAGTGACATGTATCT |
| 0800-WRKY8-R | tagaactagtggatccATATACTTGATAAGATTATCCTTTGATG |
| 62SK-PYR/PYL1-F | tagaactagtggatccATGAGCGGCAATGGATTTGG |
| 62SK-PYR/PYL1-R | cggtatcgataagcttGATCCTATCAATGGGCTCAGTC |
| 62SK-PYR/PYL2-F | tagaactagtggatccATGCCTTCAATCCAGTCATCAATT |
| 62SK-PYR/PYL2-R | cggtatcgataagcttCTTCGTTTTGGCGGCCAAAT |
| 62SK-PYR/PYL3-F | tagaactagtggatccATGCTTTCTAATCCTCCAAATTCCT |
| 62SK-PYR/PYL3-R | cggtatcgataagcttGTTTCGACGGGGCAAATTCT |
| 62SK-PYR/PYL4-F | tagaactagtggatccATGGGAAACAAGAAATGGTGTGG |
| 62SK-PYR/PYL4-R | cggtatcgataagcttCTTAAAGTTTGTGGACGAAGCTTC |

**Supplementary Table 2.** Amino acid composition of CpWRKY8 protein.

| Amino acid | Number | Percentage |
| --- | --- | --- |
| Ala (A) | 18 | 5.2% |
| Arg (R) | 16 | 4.6% |
| Asn (N) | 26 | 7.5% |
| Asp (D) | 19 | 5.5% |
| Cys (C) | 5 | 1.4% |
| Gln (Q) | 22 | 6.4% |
| Glu (E) | 18 | 5.2% |
| Gly (G) | 16 | 4.6% |
| His (H) | 6 | 1.7% |
| Ile (I) | 11 | 3.2% |
| Leu (L) | 22 | 6.4% |
| Lys (K) | 24 | 6.9% |
| Met (M) | 6 | 1.7% |
| Phe (F) | 8 | 2.3% |
| Pro (P) | 23 | 6.6% |
| Ser (S) | 43 | 12.4% |
| Thr (T) | 29 | 8.4% |
| Trp (W) | 2 | 0.6% |
| Tyr (Y) | 8 | 2.3% |
| Val (V) | 24 | 6.9% |
